# Suicide and COVID-19: a rapid scoping review

**DOI:** 10.1186/s12991-023-00441-6

**Published:** 2023-03-17

**Authors:** Tommaso Barlattani, Chiara D’Amelio, Francesco Capelli, Simonetta Mantenuto, Rodolfo Rossi, Valentina Socci, Paolo Stratta, Ramona Di Stefano, Alessandro Rossi, Francesca Pacitti

**Affiliations:** 1 Department of Biotechnological and Applied Clinical Sciences (DISCAB), University of L’Aquila, Via Vetoio, 67100 Coppito, L’Aquila Italy; 2 Department of Mental Health Sulmona-Avezzano-L’Aquila, ASL 1, Abruzzo, Italy; 3 Department of Systems Medicine, University of Rome Tor Vergata, Via Montpellier, 1, 00133 Rome, Italy

**Keywords:** Suicide, Covid-19, Mental disorder, Pandemic

## Abstract

There is considerable interest in exploring effects of coronavirus disease 2019 (COVID-19) pandemic on mental health. Suicide is one of the leading causes of mortality worldwide and changes in daily life brought by the pandemic may be additional risk factors in people with pre-existing mental disorders. This rapid PRISMA-ScR (Preferred Reporting Items for Systematic Reviews and Meta-Analyses extension for Scoping Reviews) scoping review aims to identify and analyze current evidence about the relation between COVID-19 pandemic outbreak, along with COVID-19 disease and severe acute respiratory syndrome coronavirus 2 (SARS-CoV2) infection, and suicide in individuals with previously diagnosed mental disorders. First, we conducted a comprehensive review of the literature, then proceeded to discuss findings in a narrative way. Tables were constructed and articles sorted according to the studies’ methodologies. 53 papers were eventually identified as eligible, among which 33 are cross-sectional studies, 9 are longitudinal studies, and 11 studies using other methodologies. Despite suffering from a mental disorder is a risk factor for suicidal behavior per se, the advent of COVID-19 pandemic may exacerbate this relation. Nevertheless, data addressing a clear correlation between suicidal behavior and the pandemic outbreak are still controversial. Longitudinal analysis using validated suicide scales and multicenter studies could provide deeper insight and knowledge about this topic.

## Introduction

The advent of the pandemic has drastically impacted our daily lives. At the time of writing, more than 505,560,928 million people worldwide have been infected with severe acute respiratory syndrome coronavirus 2 (SARS-CoV2), causing 6,226,457 deaths [1]. Suicide is a complex multifactorial phenomenon and a leading cause of death worldwide. Most suicides are related to psychiatric diseases, and individuals with mental disorders are at increased risk [2]. Based on the data on suicide rates relating to the previous epidemics, a rise in suicide was observed between 1918 and 1919 during the influenza epidemic in the United States [3]. These data are also consistent with increased levels of suicide among older adults during the 2003 SARS epidemic in Hong Kong [4]. During the current pandemic, extraordinary measures for treatment and prevention of infection have been put in place by governments, such as quarantines, lockdowns, and social distancing. An increase in the incidence of mental disorders such as acute stress disorder, anxiety, irritability, PTSD, elevated psychological distress, depressive symptoms, and insomnia may have been caused by the previously stated measurement [5]. All these disturbances are related to an increase in suicide risk [6]. As already demonstrated by longitudinal studies, also economic crises and unemployment have been linked to increased suicide rates [7]. Therefore, the economic downturn resulting from the advent of the coronavirus disease 2019 (COVID-19) pandemic may also be considered an additional risk factor for suicidal behavior [8]. Moreover, fear of infection, already shown as a prominent risk factor [9], social isolation, and grief may further contribute to a robust increase in suicidal behavior. SARS-CoV2 infection per se has proven to be a potential risk factor for suicidal behavior [10] and may eventually be included within COVID-19 sequelae. Furthermore, during the first phases of the pandemic outbreak, we witnessed a dramatic increase in difficulties in referring to psychiatric departments [11], along with a drastic reduction in outpatient medical care accessibility. As a result, patients with pre-existing mental disorders may have experienced an exacerbation of symptoms such as feelings of loneliness, anxiety, depression, insomnia, and hopelessness, which might eventually lead to a decrease in treatment compliance, increasing the risk of suicidal behavior [12]. Although data on previous epidemics demonstrated a correlation with increased suicide rates, the influence of the COVID-19 pandemic on suicide trends is still debated and controversial [13]. A recent meta-analysis carried out by Dubè et al. [14] highlighted how the COVID-19 pandemic had increased suicide risk in the general population; however, data regarding suicide behavior during the COVID-19 pandemic in subjects with pre-existing mental disorders are still scarce, and strong data are lacking. In this scenario, the present study aims to highlight current evidence on suicide and the COVID-19 pandemic, including studies also addressing this relation with SARS-CoV2 infection and the proper disease (to which we will refer as COVID-19 disease), in subjects with psychiatric disorders and to fill the gaps in the literature regarding this topic.

## Methods

A comprehensive review of the literature was carried out on Pubmed up to April 2, 2022. Considering the extensive aim of the study, we used Medical Subject Headings (MeSH) descriptors (“COVID-19”[Mesh]) AND ”Suicide”[Mesh]). To maximize the sensitivity of our study, we did not provide additional terms besides “Suicide” and “COVID-19”. Subsequently, the studies included were discussed with a narrative overview. Aiming to cover a broad literature overview and considering that strong evidence regarding suicide rates in subjects with a pre-existing mental disorder during the pandemic is still lacking, a rapid PRISMA-ScR (Preferred Reporting Items for Systematic Reviews and Meta-Analyses extension for Scoping Reviews) scoping review, following the statement guidelines for scoping reviews [15], was identified as the best method to carry out the present study [16]. Only articles published between March 2020, the declaration of the pandemic [17], and April 2022 were selected. Papers included examined suicidal behavior during the pandemic, the correlation between suicide rates and the pandemic itself in cross-sectional analyses, or changes in suicidal behavior in a longitudinal perspective; finally, other studies providing useful information on clinically observable suicidal behavior during the pandemic were included. In the present review we included not only studies that addressed suicidality in relation to COVID-19 pandemic, but also in relation to SARS-CoV2 infection and in COVID-19 disease. Only studies that included subjects with previous mental illness and assessed suicide behavior and risk were taken into consideration. Specifically, whether the sample included general population, the study only considered available data on suicide assessment in individuals with previously diagnosed mental illness. We excluded studies on topics other than suicide and COVID-19 pandemic, infection and disease, and in languages other than English. Being the present study a scoping review, the quality of studies is not necessarily addressed [18]; therefore, meta-analysis, reviews, and systematic reviews were excluded. Papers found to be purely narrative papers, editorials, books chapter, letters to editors, comments, and case reports with small samples (< 10 subjects) were excluded since they would not provide significant insight into the researched topic. Two independent reviewers screened citations for inclusion. Data extraction was conducted by one reviewer and verified by a second reviewer. Tables were then constructed, and articles were sorted out by authors, title, location of the study, sample size, nature of the sample, purpose/aim of the study, suicide assessments measures, type of publishing, time points compared/ analyzed in the study, and principal findings. In the narrative overview, studies were categorized by methodological approach (cross-sectional vs. longitudinal) and type of sample (patients vs. general population). Results were then discussed.

## Search results

The initial Pubmed search yielded a total of 502 results. Three additional titles were identified through other sources (website searching, citation tracking, and reference chaining). 10 records were excluded as not full text. The remaining 495 full-text records titles and abstracts were screened, and 135 were excluded as the article's primary focus was not the correlation between suicide and COVID-19, being therefore irrelevant to the present study's aim. Among the remaining 360 papers, 84 were excluded since they did not meet the inclusion criteria. Specifically, 79 articles were excluded as 16 were reviews, 16 case reports, 3 systematic reviews, 18 comments, 23 editorials, 2 meta-analyses, and 1 was retracted. 5 articles were excluded as being published 1 in French, 1 in Hungarian, 1 in German, 1 in Turkish, 1 in Italian. Of the resulting 276 eligible records, 223 were excluded as they did not provide adequate information about the relation between COVID-19 and suicide behavior, or the sample considered did not include subjects with a pre-existing mental condition. 53 papers were finally identified as of particular interest. The selected articles are presented in Tables 1, 2, 3, 4 and 5 and discussed in the narrative overview.

**Table 1:** Suicide and Covid-19 in subjects with pre-existing mental disorder, longitudinal studies patients’ sample.

*Characteristics of longitudinal studies using patients sample examining Covid-19 and suicide in subjects with pre‐existing mental health conditions (n* = *4)*										
*Authors, year*	Title	Location of study	Sample size	Nature of sample	Age range of the sample and gender	Purpose/aim of the study	Suicide assessment measures	Type of publishing	Time points compared/analyzed in the study	Principal findings
*Rømer et al. 2021*	Psychiatric Admissions, Referrals, and Suicidal Behavior Before and During the COVID‐19 Pandemic in Denmark: A Time‐Trend Study	Denmark, Capital Region of Denmark and Region Zealand	2,693,924 health records	Health records from hospitals and Emergency Medical Services. With regard to suicide attempts, self-harm episodes and suicides registered at hospitals in The Capital Region of Denmark and Region Zealand between 2019–2021 subjects with pre-existing mental disorders were: 829 in 2019 (76.8%); 770 in 2020 (76.3%); 224 in 2021 (83.3%)	Age range of the sample with pre-existing mental disorder: in 2019: between 0–17: 262, between 18–29: 238, > 30: 355; in 2020: between 0–17: 247, between 18–29: 221, > 30: 344; in 2021 (Jan–Feb) between 0–17: 108, between 18–29: 54, > 30: 63; female in 2019: 676 (62.6%), female in 2020: 648 (64.2%), female in 2021 (Jan–Feb) 190 (70.6%)	To assess the patterns in psychiatric admissions, referrals, and suicidal behavior before and during the COVID‐19 pandemic	Diagnoses in the electronic health records (EHRs)—including codes for suicide and self‐harm—defined and coded according to the ICD‐10 system by the responsible clinicians	Population‐based study, Longitudinal study	From January 1, 2016 to February 28, 2021. Regarding pandemic during the first lockdown (March 11, 2020 – May 17, 2020), the inter‐lockdown period (May 18, 2020–December 15, 2020), and the second lockdown (December 16, 2020 –February 28, 2021)	Most patients exhibiting suicidal behavior had pre‐existing mental disorders. The hospital‐registered rate of suicidal behavior events during the pandemic did not change significantly compared to the pre‐pandemic period; nor did it change during the first lockdown, the inter‐lockdown period or second lockdown. This pattern was observed for all people with pre-existing mental disorder. Moreover, trend in hospital‐recorded suicidal behavior during the pandemic as a whole showed a relative decline compared with the pre‐pandemic trend among patients with pre‐existing mental disorders. In particular, the relative change in rate ratio regarding suicidal behavior during the pandemic vs. pre-pandemic shows a statistically significant decrease in subjects with mood disorder and neurotic, stress-related and somatoform disorders
*Khosravani *et al*. 2021*	The associations of obsessive–compulsive symptom dimensions and general severity with suicidal ideation in patients with obsessive–compulsive disorder: The role of specific stress responses to COVID-19	Shahid Beheshti University of Medical Sciences, Teheran, Iran	390 OCD patients	Patients referred for treatment of a primary OCD diagnosis. Common comorbid disorders were major depressive disorder (MDD), bipolar disorder (BD), various anxiety disorders, and substance use disorders (SUDs)	Mean age of the sample: 35.8 years; 126 males, 178 females	To examine the effects of obsessive–compulsive (OC) symptom dimensions and OCD severity on suicidal ideation by considering the role of stress responses in reaction to COVID‐19 in a clinical sample of patients with OCD	Suicide risk assessed using Beck Scale for Suicidal Ideation (BSSI)	Longitudinal study	Between 5 June to 30 October 2020	The obsessive–compulsive symptom dimensions of responsibility for harm and unacceptable obsessional thoughts as well as general severity had indirect effects on suicidal ideation through the specific stress responses to COVID-19, including traumatic stress and compulsive checking. The study shows that OCD patients with specific obsessive–compulsive symptom dimensions and severe OCD are more likely to have suicidal ideation during the pandemic
*Alonso* et al*. 2021*	How is COVID-19 affecting patients with obsessive–compulsive disorder? A longitudinal study on the initial phase of the pandemic in a Spanish cohort	Hospital de Bellvitge, Barcelona, Spain	364; 127 OCD patients, 237 controls	Participants from the general population recruited through social networks, using a snowball method Adult outpatients who had been attending the specialist for at least one year before March 2020	Mean age of the sample: 42.0 years OCD patients; 40.8 years controls	To evaluate the impact of the COVID-19 pandemic on a sample of patients with OCD at the initial stage of the health crisis, assessing not only changes in OCD severity, but also in pre-existing conditions, newly developed conditions, treatment, use of mental health resources, development of obsessive fears of SARS-CoV-2 contamination, and use of emotional regulation and stress coping strategies	Suicidal ideation assessed according to the Hamilton Depression Rating Scale (HDRS; item on suicide)	Naturalistic cohort study, Longitudinal study	From April 27 to May 25, 2020	Suicide-related thoughts were more frequent among the OCD cohort than among healthy controls. The current crisis constitutes a risk factor for a significant worsening of symptoms and suicidal ideation
*Na *et al*. 2021*	Prevalence, risk and protective factors associated with suicidal ideation during the COVID-19 pandemic in U.S. military veterans with pre-existing psychiatric conditions	USA	661 veterans	Data analyzed from the National Health and Resilience in Veterans Study, which surveyed a nationally representative cohort of U.S. veterans Veterans screened positive for major depressive disorder (MDD), generalized anxiety disorder (GAD), post-traumatic stress disorder (PTSD), and/or substance use disorder (SUD) at the pre-pandemic assessment	Mean age of the sample: 55.2 years; 86.8%, male	To examine pre-pandemic, COVID-related, and changes in risk and protective factors associated with peri-pandemic suicidal ideation. To evaluate interactions between SARS-CoV2 infection and age, and significant protective factors, in predicting suicide ideation in the examined population	Suicidal ideation assessed using Patient Health Questionnaire-9 (PHQ-9)	Prospective, Longitudinal survey cohort, Longitudinal study	Pre-pandemic survey: until 11/21/2019 peripandemic survey: until 11/14/2020	Those who were infected with SARS-CoV 2 and aged 45 or older or who reported lower purpose in life may be at the highest risk of suicide and may deserve close clinical attention and monitoring

**Table 2:** Suicide and Covid-19 in subjects with pre-existing mental disorder, longitudinal studies general population.

*Characteristics of longitudinal studies using general population examining Covid-19 and suicide in subjects with pre‐existing mental health conditions (n* = *5)*										
*Authors, year*	Title	Location of study	Sample size	Nature of sample	Age range of the sample and gender	Purpose/aim of the study	Suicide assessment measures	Type of publishing	Time points compared/analyzed in the study	Principal findings
*Batterham *et al*. 2022*	Effects of the COVID-19 pandemic on suicidal ideation in a representative Australian population sample–Longitudinal cohort study	Australia	1296 subjects	Nationally representative sample of Australian adults. Regarding history of mental illness 246 (19.0%) had a past diagnosis 310 (23.9%) had a current diagnosis	Mean age of the sample: 46.0 years; 50.1% female	To assess the prevalence of suicidal ideation in a representative population-based sample in the first 12 weeks of the COVID-19 pandemic in Australia, to assess the persistence of suicidal ideation using long-term follow-up data, and, to assess relationships between indirect effects of the pandemic (financial, social, employment) on suicidal ideation, while accounting for demographic factors	Suicidal ideation assessed according to the suicidal item of Patient Health Questionnaire, PHQ-9	Longitudinal survey	From late-March to June 2020	Current diagnosis of mental illness was associated with double the risk of suicidal ideation, while past diagnosis was associated with a 38% increase in risk. However, past mental health diagnosis was not significantly associated with incident suicidal ideation
*Fountoulakis *et al*. 2021*	Results of the COVID-19 mental health international for the general population (COMET-G) study	40 countries: Argentina, Australia, Azerbaijan, Bangladesh, Belarus, Brazil, Bulgaria, Canada, Chile, Croatia, Egypt, France, Georgia, Germany, Greece, Honduras, Hungary, India, Indonesia, Israel, Italy, Japan, Kyrgyz Republic, Latvia, Lithuania, Malaysia, Mexico, Nigeria, Pakistan, Peru, Poland, Portugal, Romania, Russia, Serbia, Spain, Turkey, Ukraine, UK, USA	55,589 subjects from 40 countries	International general population sample Any mental disorder history was present in 25.25% of the sample 7.85% had a prior history of an anxiety disorder, 12.57% of depression, 1.16% of bipolar disorder, 0.97% of psychosis and 2.70% of other mental disorder. At least once, 21.44% had hurt themselves in the past and 10.59% had attempted at least once in the past	Mean age of the sample: 35.80 years females, 34.90 years males, other 31.64 years; 64.85% females, 34.05% males and 1.10% other	To investigate the rates of distress, probable depression and suicidality and their changes in the adult population aged 18–69 internationally, during the COVID-19 pandemic	Suicide risk assessed using Risk assessment suicidality scale (RASS)	Multiple Forward Stepwise Linear Regression Analysis, Longitudinal study	In 2019, data collected from June 3 through July 12 In 2020, data collected from August 3 through November 13	Multiple forward stepwise linear regression analysis revealed that a broad number of variables acted either as risk or as protective factors accounting for the 4,7% change in suicidal behavior. Suffering from a previous mental condition acted as a risk factor and suicidal behavior resulted increased in those people during pandemic
*Nichter* *et* *al. 2021*	Prevalence and Trends in Suicidal Behavior Among US Military Veterans During the COVID-19 Pandemic	USA	3078 US veterans	Population-based cohort of US military veterans In the no suicide ideation group: 387 (16,3%) have a lifetime post-traumatic stress disorder (PTSD) and/or major depressive disorder (MDD), 1017 (39,3%) have alcohol use disorder (AUD) and/or drug use disorder (DUD) In the new-onset suicide ideation group: 34 (52.9%) have a lifetime post-traumatic stress disorder (PTSD) and/or major depressive disorder (MDD), 52 (63.8%) have alcohol use disorder (AUD) and/or drug use disorder (DUD)	Mean age of the sample: 63.2 years; mostly male 2734 (91.6%)	To examine longitudinal changes in suicidal behavior from before the COVID-19 pandemic to nearly 10 months into the pandemic and identify risk factors and COVID-related variables associated with new-onset suicide ideation (SI)	Suicide behavior assessed trough the Suicidal Behaviors Questionnaire-Revised (SBQ-R)	Population-based prospective cohort study, Longitudinal study	From November 18, 2019, to December 19, 2020	Rates of suicide ideation and suicide attempts did not significantly increase from pre-pandemic to peripandemic at the population level. However, a small proportion of veterans (2.6%) developed new-onset suicide ideation during the pandemic Among the strongest risk factors and COVID-19-related variables for new-onset suicide ideation were suicide attempt history, lifetime post-traumatic stress disorder and/or depression, and past-year alcohol use disorder severity
*Na *et al*. 2021*	Mental health and suicidal ideation in US military veterans with histories of COVID-19 infection	USA	3078 veterans	Nationally representative, prospective cohort of US veterans. 233 veterans (8.6%) reported having been infected with SARS-CoV2. Relative to veterans who were not infected, veterans who were infected were more likely to screen positive for internalizing disorders (major depressive disorder, generalized anxiety disorder and/or pandemic-related stress symptoms) 20.5% vs 13.9%, externalizing disorders (alcohol and/or drug use disorder) 23.2% vs 14.8% and current suicidal ideation 12.0% vs 7.6% at peripandemic	Mean age of the sample: 62.2 years; the majority was male (90.2%)	To date, the prevalence, risk and protective factors of psychiatric conditions among US military veterans who survived COVID-19	Suicidal ideation assessed according to the suicidal item of Patient Health Questionnaire, PHQ-9	Prospective, Longitudinal survey cohort, Longitudinal study	From pre-pandemic survey (median completion date: 21 November 2019), to peripandemic 1-year follow-up assessment (median completion date 14 November 2020)	Pre-pandemic alcohol use severity, past-year suicidal ideation, loneliness, impulsivity, perceived social support and having a household member infected with SARS-CoV2 were independent risk factors for peripandemic suicidal ideation, whereas greater protective psychosocial characteristic Greater pre-pandemic psychiatric symptoms severity, were independent risk factors for peripandemic internalizing psychiatric disorders
*Fountoulakis *et al*. 2021*	Self-reported changes in anxiety, depression and suicidality during the COVID-19 lockdown in Greece	Greece	3399 subjects	Nationwide representative sample of the general population. History of any mental disorder reported by 29.60%, with history of depression being the most frequent (26.92%) Psychotic disorders (0.49%), Bipolar disorder (0.12%), Eating disorders (0.11%) and Substance abuse disorder (0.02%) were rather rare, but within the expected range	Mean age of the sample: female 34.02 years, males 36.38 years; 81.08% females, 18.27% males	To investigate the rate of clinical depression in the adult population aged 18–69 in Greece, during the period of the lockdown. To investigate the changes in anxiety, distress, suicidal ideation and their relations with a number of personal and interpersonal/social variables. The aim also included the investigation of the spreading of conspiracy theory beliefs concerning the COVID-19 outbreak	Suicide risk assessed using Risk assessment suicidality scale (RASS)	Multiple Forward Stepwise Linear Regression Analysis, Longitudinal study	From April 11th to May 1st, 2020	Suicidal thoughts increased in 10.40% and decreased in 4.42%. Comparison of cases without vs those with a previous history of depression in terms of the changes in suicidal thoughts suggested that the two groups differed in any increase in suicidal ideation (8.39% vs. 15.66%). Comparison of the numbers of cases without vs. those with a previous history of suicide attempts in terms of changes in current suicidal ideation suggested that the two groups differed in any increase in suicidal ideation (9.96% vs. 23.19%). Previous history of depression, self-harm and suicidal attempts act a risk factors in the develop of depression and, eventually, to suicidality

**Table 3:** Suicide and Covid-19 in subjects with pre-existing mental disorder, cross-sectional studies patients’ sample

*Characteristics of cross-sectional studies including patients’ sample, examining Covid-19 and suicide in subjects with pre‐existing mental health conditions (n* = *12)*										
*Authors,* *year*	Title	Location of study	Sample size	Nature of sample	Age range of the sample and gender	Purpose/aim of the study	Suicide assessment measures	Type of publishing	Time points compared/analyzed in the study	Principal findings
*Liu *et al*. 2022*	Prevalence of Suicidality and its Association with Quality of Life in Older Patients with Clinically Stable Psychiatric Disorders in China During the COVID-19 Pandemic	China	1063 patients	Clinically stable psychiatric patients. Patients with major depressive disorder were 485 (45.6%); patients with Bipolar disorder were 43 (4%); patients with Schizophrenia were 73 (6.9%)	Mean age of the sample: 62.80 years; 347 male (32.6%)	To examine the prevalence of suicidality and its association with quality of life (QOL) among older clinically stable patients with psychiatric disorders during the COVID-19 pandemic	Suicidality during the COVID-19 outbreak was evaluated by 3 “yes” or “no” standardized questions, including (1) suicidal ideation, (2) suicide plan and (3) suicide attempt. Patients who responded “yes” to any of the 3 questions considered “having suicidality.”	Multicenter, cross-sectional study	From May 22 to July 15, 2020	The prevalence of suicidality was 11.8% during the COVID-19 pandemic. Suicidality was common in older patients with clinically stable psychiatric disorders during the COVID-19 pandemic. Of the patients with suicidality, major depressive disorder was the most common psychiatric diagnosis
*Kang* et al*. 2021*	Changes in the pattern of suicide attempters visiting the emergency room after COVID-19 pandemic: an observational cross-sectional study	Busan University Hospital, South Korea	879 Emergency department patients records	Busan University Hospital emergency room medical records. Patients with previous psychiatric history were 163 (40.8%) in the Pre-pandemic group and 381 (86.6%) in the Pandemic group	Mean age of the sample: 40.68 years Pre-pandemic group, 40.90 years Pandemic group; Female were 250 (62.5%) in the Pre-pandemic group, 283 (64.30%) in the Pandemic group	To find out the change in the rate and pattern of suicide attempts during severe acute respiratory syndrome in COVID-19 pandemic period	Data collection including, history of mental illness; and suicide attempt, suicide method, and location (i.e., at home or a place other than home) at the time of attempt, and whether the attempt was a mass suicide. Severity of suicide attempt was assessed trough the South Korean triage and acuity scale (KTAS)	Retrospective, Cross-sectional study	Pre-pandemic (January 19 to October 31, 2019) and during pandemic (January 19 to October 31, 2020)	The number of patients who had a history of psychiatric treatment was 163 (40.8%) during the “pre-COVID-19 period” and 381 (86.6%) during the “COVID-19 period”. The increase in the number of patients with a history of psychiatric treatment due to suicide attempts can be interpreted as a result of increased anxiety and depression caused by the lack of mental health treatment. Severity of patients who visited the emergency room following a suicide attempt was higher during the “COVID-19 period”
*Grossman* et al*. 2021*	Trends in suicidal ideation in an emergency department during COVID-19	Academic medical center in Boston, Massachusetts, USA	339 emergency department patients in the “comparison series” and 216 emergency department patients in the “COVID-19 series”	Patients presenting with suicidal ideation to a consult liaison service. During December 2018 – February 2019 (comparison pre-period series) % with any psychotic disorder were 7.1%, % with any affective disorder were 39.1%, % with substance use disorder were 50.0% During December 2019 – February 2020 (COVID-19 pre-period series) % with any psychotic disorder were 3.2%, % with any affective disorder were 45.5%, % with substance use disorder were 48.4%	Mean age of the sample: 36 years in the “comparison series”, 38 years in the “COVID-19 series”; female 42.2 (2.9%) in the “comparison series”, 37.2 years (2.9%) in the “COVID-19 series”	To detail changes in presentations at a United States Emergency Department for suicidality before and after the outbreak of COVID-19	Data on suicidality characteristics pulled from notes in the record, regarding patients presenting with suicidality in the emergency department, and received emergency psychiatric consultation	Retrospective, Cross-sectional study	Between December 2018 – May 2019 and December 2019 – May 2020	Pre-period differences did show a lower proportion of presentations for suicidality among people with any psychotic disorder in the COVID-19 pre-period cohort relative to the comparator cohort. Patients visits in the COVID-19 post-period were less likely to be attributed to psychiatric symptoms as a reason for suicidality compared to visits in the comparator post-period (70.5% vs. 50.0%; *p*-value < 0.001). Conversely, there were also differential increases in the proportion of patients visits to the emergency department with a history of prior suicide attempts in the COVID-19 period relative to the comparison period (13.2 percentage points and 13.0 percentage points, respectively). No significant differences were found between the groups among patients with coexisting psychotic disorder, anxiety disorder, and substance abuse disorder with regard to suicidality presentation in the emergency department during the time considered
*Ridout et* *al. 2021*	Emergency Department Encounters Among Youth with Suicidal Thoughts or Behaviors During the COVID-19 Pandemic	Kaiser Permanente Northern California, USA	2123 youth patients in 2020, 2339 youth patients in 2019	Youth aged 5 to 17 years with suicide-related ED encounters	Age range of the sample: individuals aged 13 to 17 years: 1798 (84.7%) in 2020, 1998 (85.4%) in 2019; 1483 female (69.9%) in 2020, 1542 female (65.9%) in 2019	To characterize population-level and relative change in suicide-related ED encounters among youth during the COVID-19 pandemic compared with 2019	Population-level incidence rate ratios (IRRs) and percent relative effects for suicide-related ED encounters as defined by the US Centers for Disease Control and Prevention recommended (ICD-10-CM)	Retrospective, Cross-sectional study	Pre-pandemic (January 1, 2019, to December 15, 2019) and during pandemic (January 1, 2020, to December 15, 2020)	Youth with no history of outpatient mental health or suicide encounters and those with comorbid psychiatric conditions documented at the emergency department encounter had a higher risk of presenting with suicide-related problems from September to December 2020 (pandemic) versus the same period in 2019. There was a 6.7% higher risk of having a comorbid psychiatric nonsubstance diagnosis at the time of the suicide-related ED encounter during the fall compared with 2019 levels
*Lee et. al 2021*	Association of the COVID-19 Pandemic and Low-rescue Suicide Attempts in Patients Visiting the Emergency Department after Attempting Suicide	St. Mary's Hospital, Seoul, South Korea	518 subjects	Patients who made a suicide attempt and visited the emergency department. Subjects with previous psychiatric history were 274 (52.9%)	Mean age of the sample: 38 years; 205 patients (39.6%) were male	To investigate whether the factors affecting the lethality of suicide attempts differed before and during the COVID-19 pandemic using the RRRS in patients who attempted suicide and visited the emergency department	Data on characteristics of the suicide attempt and about the patients from the medical and counseling records. Suicide lethality assessed using the Risk-rescue rating in suicide assessment (RRRS)	Retrospective, Cross-sectional study	From March 2019 to September 2020	Suicide attempts associated with mental disorders were more common in the before COVID-19 group (P < 0.001). COVID-19 pandemic was an independent risk factor for low-rescue suicide attempts. History of previous suicide attempts and previous psychiatric history were not significant independent factors for low-rescue suicide attempts
*Berardelli* *et. al 2021*	The impact of the COVID-19 pandemic on suicide ideation and suicide attempts in a sample of psychiatric inpatients	Sant'Andrea University Hospital, Rome, Italy	632 Psychiatric patients’ clinical records; 315 before the lockdown, 317 during Covid-19 pandemic	Clinical records of psychiatric patients admitted to a public psychiatric clinic. Among the whole sample 22.9% has Bipolar disorder, 13.9% Depressive disorder, 29.4% Schizophrenia and psychoses, 10.4% Personality disorders	Mean age of the sample: 42.25 years; 311 women and 321 men	To see whether the frequency of suicide ideation and suicide attempts differed in psychiatric patients before and during the COVID-19 pandemic and government lockdown restrictions	Suicide attempt investigated at the time of arrival of the patient at the emergency department; suicide ideation investigated according to the definition in the Columbia–Suicide Severity Rating Scale (C-SSRS)	Retrospective, Cross-sectional study	Between May 2019 and December 2020	Only suicide attempts, but not suicide ideation, were more frequent in psychiatric patients admitted during the COVID-19 pandemic than before
*Mutlu et* *al. 2021*	Relapse in patients with serious mental disorders during the COVID-19 outbreak: a retrospective chart review from a community mental health center	Community mental health centers Etimesgut, Ankara, Turkey	155 Psychiatric patients	Medical charts of psychiatric patients 131 with schizophrenia/ schizoaffective disorder, 24 with bipolar disorder	Mean age of the sample: 46.6 years; 68% male	To investigate the basic characteristics of patients who experienced relapse during the first trimester (from 10th of March to 10th of June) of the COVID-19 outbreak in Turkey, and to compare main findings with the same period in 2019	Relapse criteria defined including: new onset of suicidal thoughts or suicide attempt, non-suicidal self-harm	Retrospective, Cross-sectional study	Pre-pandemic (from 10th of March to 10th of June 2019) and during pandemic (from 10th of March to 10th of June 2020)	The relapse rate of the patients in the first trimester of COVID-19 outbreak was 11% (2 bipolar disorder, 15 schizophrenia). 2 had new onset of suicidal thoughts/suicide attempt, and 3 showed self-harm or violent behavior. The relapse rate of the sample in 2019 was 6.5% (1 bipolar disorder, 9 schizophrenia or schizoaffective disorder), and did not differ from the first trimester of COVID-19
*Seifert* et al*. 2021*	Peripandemic psychiatric emergencies: impact of the COVID-19 pandemic on patients according to diagnostic subgroup	Hannover Medical School, Hannover, Germany	750 374 in 2020; 476 in 2019	Patients presenting in the psychiatric emergency department In 2020, substance use disorders were 114 (30.5%); schizophrenia, schizotypal, and delusional disorders were 70 (18.7%); affective disorders were 57 (15.2%); neurotic, stress-related, and somatoform disorders were 70 (18.7%); personality and behavioral disorders were 46 (12.3%); others were 17 (4.5%) In 2019, substance use disorders were 138 (29.0%); schizophrenia, schizotypal, and delusional disorders were 92 (19.3%); affective disorders were 106 (22.2%); neurotic, stress-related, and somatoform disorders were 76 (16%); personality and behavioral disorders were 37 (7.8%); others were 27 (5.7%)	Mean age of the sample: 43.4 years in 2020, 44.48 years in 2019; 147 females (39.3%) in 2020, 228 females (47.9%) in 2019	To detect the impact of the COVID-19-pandemic on patients within different psychiatric diagnostic subgroups presenting in the psychiatric emergency department	Psychopathological assessment (PPA) according to the “Arbeitsgemeinschaft für Methodik und Dokumentation in der Psychiatrie” (AMDP)-System (including suicidality)	Retrospective, Cross-sectional study	Between March 16th and May 24th 2020; Between March 16th and May 24th 2019	The rate of patients stating suicidal ideation (32.9 vs. 29.6%) and intent (12.3 vs. 9.9%) remained stable between 2019 and 2020 Suicidal ideation stated significantly more often by patients with substance use disorders in 2020 than in 2019. Patients with schizophrenia presenting during the COVID-19 pandemic did not differ in suicidal ideation/intent. Patients stating an association with COVID-19 were nearly three times more likely to have attempted suicide prior to presentation in the psychiatric emergency department compared to the overall rate of suicide attempts leading to presentation in the psychiatric emergency department
*Montalbani *et al*. 2021*	The COVID-19 Outbreak and Subjects with Mental Disorders Who Presented to an Italian Psychiatric Emergency Department	Sant'Andrea University Hospital, Rome, Italy	371 Records for 213 Patients	Patients with psychiatric disorders who presented for psychiatric counseling 57 had major depressive disorder, 36 bipolar disorder, 56 had generalized anxiety disorder, and 27 patients had schizophrenia and other psychotic disorders 9 had a full-blown personality disorder, and the remaining had other diagnoses	Mean age of the sample: 42.9 years, Range 18–86; Men 97	To assess the socio-demographic and clinical features of patients who required a psychiatric consultation in the emergency department of an Italian hospital during the COVID-19 outbreak	Suicide risk assessed with the Columbia Suicide Severity Rating Scale (C-SSRS)	Retrospective, Cross-sectional study	Between January 1 and May 3, 2020 Before lockdown measures until the 11^th^ of March compared with after lockdown measures, thus after the 11^th^ of March	Patients who presented during the lockdown showed greater active suicidal ideation in terms of intentionality and planning. People with mental issue may have experienced an increase in symptoms during pandemic
*Jefsen* et al*. 2021*	COVID‐19‐related self‐harm and suicidality among individuals with mental disorders	Psychiatric services of the Central Denmark Region, Denmark	102 Clinical notes from 74 Psychiatric patients	Clinical notes from the adult psychiatric services. Regarding diagnosis: 12 have Schizophrenia and other psychotic disorders, 10 have Mood disorders, 13 have Stress‐related and adjustment disorders, 14 have Personality disorders, 5 have Autism, 15 Other diagnosis	Mean age of the sample: 29.8 years; 77% were females	Deeper thorough characterization of suicide behavior, detection, and care of patients with pandemic‐related psychopathology	Clinical notes divided according to diagnosis and divided into five different categories according to suicide behavior: 1—thoughts of self‐harm, 2—completed self‐harm, 3—passive wish to die of COVID-19, 4—suicidal thoughts, 5—suicide attempts	Retrospective, Cross-sectional study	From February 1st to March 23rd 2020	There is known ‘high risk’ groups for self‐harm and suicidality composed by: psychotic disorders, mood disorders, stress‐related and adjustment disorders, and personality disorders, which appear to respond to the stress associated with the COVID‐19 pandemic with these symptoms/behaviors. COVID‐19 crisis led to increased self‐harm/suicidality in individuals with mental disorders
*Menculini* et al*. 2021*	Suicidality and COVID-19: Data from an Emergency Setting in Italy	General Hospital of Perugia, Perugia, Italy	447 patients	Patients requiring psychiatric consultations carried out at the emergency department 109 subjects were assessed for suicidality. Regarding diagnosis: 23 had affective disorders (21,1%), 15 schizophrenia spectrum disorders (13,8%), 8 anxiety disorders (7,3%), 6 adjustment disorders (5,5%), 9 substance-related and addictive disorders (8,3%), 1 trauma-related disorders (0,9%), 2 obsessive–compulsive and related disorders (1,8%), 24 personality disorders (22%)	Mean age of the sample: 42.44 years, 45.42 years for suicide attempt (SA), 43.28 years for suicide ideation (SI), 39.29 years for non-suicidal self-injury (NSSI); subjects included were mainly females 63 (57.8%)	To analyze the prevalence of suicidality-related phenomena during the COVID-19 pandemic among people requiring a psychiatric consultation in an emergency setting	For suicidality, data concerning suicide attempt (SA), suicide ideation (SI), and non-suicidal self-injury (NSSI) were registered	Retrospective, Cross-sectional study	From June 1st, 2020 to January 31st, 2021	A statistically significant association was detected between suicidality-related phenomena and adjustment disorders (p = 0.018). More than one-third of the sample did not report previous psychiatric history. This suggests that a percentage of cases was related to the new onset of suicidality, which is contrasting with previous findings that demonstrated how suicide-related phenomena mainly emerge in subjects affected by serious psychiatric disorders
*Almaghrebi *et al*. 2021*	Risk factors for attempting suicide during the COVID-19 lockdown: Identification of the high-risk groups	King Saud Medical City (KSMC), Riyadh, Saudi Arabia	29 suicide patients	Patients aged ≥ 16 years who survived the suicide attempts admitted to King Saud Medical City 24.1% of the suicide attempters have a history of using alcohol or drugs (7), 31% have psychosis or loss of rational thinking (9), 72.4% have Depression or hopelessness (21), 69% have previous suicide attempt or psychiatric care (20)	Age range of the sample: < 19 or > 45 years: 13 (44.8%), 19–45 years 16 (55.2%); Male 10 (34.5%) Female 19 (65.5%)	To identify the suicide-related risk factors and stressors and to determine the groups at a greater risk of attempting suicide during the COVID-19 lockdown	Modified SAD PERSONS scale (MSPS) to evaluate the suicide risk factors	Retrospective, Cross-sectional study	From April to June, 2020	Patients with psychiatric disorders accounted for 69% of the cohort. Factors like hopelessness and depression were highly related to suicide attempts, as well as the statement of future intent to repeat the attempt, at 72.4% and 65.5%, respectively. Patients with pre-existing psychiatric disorders carry high risk of attempting suicide during the COVID-19 lockdown

**Table 4:** Suicide and Covid-19 in subjects with pre-existing mental disorder, cross-sectional studies general population

*Characteristics of cross-sectional studies using general population sample, examining Covid-19 and suicide in subjects with pre‐existing mental health conditions (n* = *21)*										
*Authors*, *year*	Title	Location of study	Sample size	Nature of sample	Age range of the sample and gender	Purpose/aim of the study	Suicide assessment measures	Type of publishing	Time points compared/analyzed in the study	Principal findings
*Sáiz *et al*. 20 2022*	Suicidal Ideation Trends and Associated Factors in Different Large Spanish Samples During the First Year of the COVID-19 Pandemic	Spain	Survey 1 (April 16–22, 2020) total sample: 6,108; Survey 2 (October 14 – November 8, 2020) total sample: 6,418; Survey 3 (March 16–31, 2021) Total sample: 5,654	Spanish general population sample. In the Survey 1 group 786 (12.9%) had a past mental disorder and 510 (8.3%) had a current mental disorder. In the Survey 2 group 914 (14.2%) had a past mental disorder and 1,490 (23.2%) had a current mental disorder. In the Survey 3 group 606 (10.7%) had a past mental disorder and 704 (12.5%) had a current mental disorder	Age range of the sample: Survey 1 group 45.78 years, Survey 2 group 34.71 years, Survey 3 group 39,65 years; 4280 female (70.1%) Survey 1 group; 5731 female (89.3%) Survey 2 group, 4575 female (80.9%) Survey 3 group	To determine the prevalence of passive SI (PSI) and active SI (ASI) in Spanish general population surveys conducted at 3 points in time during the COVID-19 pandemic and to characterize the main factors associated with ASI	Suicide behavior assessed using Paykel Suicide Scale (PSS) Passive suicidal ideation (PSI) defined as positive answers to PSS items 1 and/or 2, and active suicidal ideation (ASI), as positive answers to PSS items 3 and/or 4	Cross-sectional survey	Between April 16–22, 2020 peak of first wave; between October 14–November 8, 2020 peak of second wave; between March 16–31, 2021 peak of third wave	Personal history of suicide attempt, current or past history of mental disorder are consistent risk factors for active suicidal ideation (ASI)
*Sasaki *et al*. 2022*	Temporary employment and suicidal ideation in COVID‐19 pandemic in Japan: A cross‐sectional nationwide survey	Japan, University of Tokyo Graduate School of Medicine	12 249 individuals	Nationally representative cross‐sectional study in Japan. Subjects with psychiatric history were 1636 (13.4%)	Mean age of the sample: 43.3 years; 5154 Female (42.1%)	To assess the association between employment contract and suicidal ideation or newly developed under COVID‐19 pandemic examined using a nationally representative cross‐sectional study in Japan	Suicidal ideation measured using one item: “Have you ever wanted to die from April 2020 to the present?” The response options were “1. Experienced for the first time,” “2. It has been around for a long time,” and “3. Not”. Persistent suicidal ideation that began prior to the pandemic defined as Yes (2). Newly developed suicidal ideation in the COVID‐19 pandemic defined as Yes (1)	Retrospective, Cross‐sectional study	From August to September 2020	In the subjects with previous psychiatric history group 386 (23.6%) reported persistent suicidal ideation and 146 (8.9%) reported newly developed suicidal ideation in the COVID‐19 pandemic. History of psychiatric disease was associated with newly developed suicidal ideation in COVID‐19 pandemic and persistent suicidal ideation
*Steinmetz* et al*. 2020*	Levels and predictors of depression, anxiety, and suicidal risk during COVID-19 pandemic in Argentina: the impacts of quarantine extensions on mental health state	National University of Córdoba, Córdoba, Argentina	1202 subjects	Argentineans who took part to an online survey. Subjects with mental disorder history were 302 (25.13%)	Mean age of the sample: 31.45 years; 1029 female (85.61%)	To analyze differences in mental health state (MHS) indicators (including suicidal risk), during three quarantine sub-periods; assess multiple relationships between each MHS indicator and potentially affecting factors	Suicidal risk assessed with Inventory of suicide Orientation (ISO-30)	Cross‐sectional survey	From the 30th March until 23 May 2020	This study suggests a negative mental health impact of quarantine in students and in the general population Suicidal risk, increasing from the first to the second/third quarantine extensions, but then maintaining to the fourth extension Presence of mental disorder history, and suicide attempt history were predictors of suicidal risk during quarantine sub-periods
*Mortier *et al*. 2021*	Thirty-day suicidal thoughts and behaviors in the Spanish adult general population during the first wave of the Spain COVID-19 pandemic	Spain	3500 subjects	Nationally representative sample of non-institutionalized Spanish adults. Among the sample 34.3% of respondents had pre-pandemic lifetime mental disorders: 490 have depression, 55 have bipolar disorder, 199 have panic attacks, 1052 have anxiety, 37 have alcohol use problems, 50 have drug use problems	Mean age of the sample: 49.6 years; 51.5% female	To investigate the prevalence of suicidal thoughts and behaviors (STB; suicidal ideation, plans or attempts) in the Spanish adult general population during the first wave of the Spain coronavirus disease 2019 (COVID-19) pandemic (March–July, 2020), and to investigate the individual- and population-level impact of relevant distal and proximal STB risk factor domains	Suicidal risk assessed with a modified version of selected items from the Columbia Suicide Severity Rating Scale	Cross-sectional survey	From 1–30 June 2020	STB was 9.7% among the 34.3% of respondents with pre-pandemic lifetime mental disorders, and 1.8% among the 65.7% without any pre-pandemic lifetime mental disorder. Among factors significantly associated with STB were pre-pandemic lifetime mental disorders and current mental disorders. Individual-level impact was particularly high for bipolar disorder while population-level impact was highest for depression and anxiety. About 49.1% of any STB is potentially attributable to the joint effects of all pre-pandemic lifetime mental disorders. 74.1% of suicidal thought and behavior is potentially attributable to mental disorders and adverse events—experiences related to the pandemic. Taken together these two observations suggest a potential increase of suicidal thought and behavior during the pandemic
*Vrublevska *et al*. 2021*	Factors related to depression, distress, and self-reported changes in anxiety, depression, and suicidal thoughts during the COVID-19 state of emergency in Latvia	Latvia	2608 respondents	Nationwide representative sample of the general population. 7.82% overall self-reported history of depression 6.13% had an history of at least one suicide attempt	Mean age of the sample: men 48.04 years, women 44.74 years; 1260 men, 1344 women, 4 respondent reported that they were ‘other’ or did not want to define their gender	To investigate the impact of the COVID- 19 pandemic on the mental health of the general population of Latvia	The Risk Assessment of Suicidality Scale (RASS) used to assess suicidal behavior	Cross-sectional survey	From 6 to 27 July 2020	Suicidal thoughts increased in 13.30% of those with a history of clinical depression, and 27.05% of those with a history of suicidal attempts during the state of emergency
*Papadopoulou *et al*. 2021*	Suicidal ideation during COVID-19 lockdown in Greece: Prevalence in the community, risk and protective factors	Greece	5,748 subjects	Adults who participated in the survey Mental health history, reported by 464 patients In the non-suicidal ideation group 399 (8.3%) reported mental health history In the suicidal ideation group 65 (24.4%) reported mental health history	Age range of the sample: Non-suicidal ideation group: 18–24 years: 653 (13.13%), 25–34 years: 930 (19.23%), 35–44 years: 1376 (28.45%), 45–54 years: 1148 (23.74%), 55–64 years: 618 (12.78%) 65 years and above: 129 (2.67%); Suicidal ideation group: 18–24 years: 71 (26.69%), 25–34 years: 60 (22.56%), 35–44 years: 60 (22.56%), 45–54 years: 56 (21.05%), 55–64 years: 16 (6.02%), 65 years and above: 3 (1.13%); 1,434 males, 4,217 females and 5 individuals who reported “other” sex	To investigate the prevalence of suicidal ideation in the community as well as the risk and protective factors of suicidal ideation during restriction measures in Greece, after the outbreak of the COVID- 19 pandemic and development of mental health symptoms	Suicidal ideation assessed according to the suicidal item of Patient Health Questionnaire, PHQ-9	Cross‐sectional survey	From April 7 to May 3, 2020	Individuals with suicidal ideation compared to those without suicidal ideation were more likely to have a mental health history, poorer perceived quality of physical health, and belong to a high-risk group for SARS-CoV2 infection. Participants with a mental health history had 1.64-fold higher odds of suicidal ideation Mental health history emerged among the risk factors of suicidal ideation
*Wathelet *et al*. 2020*	Factors Associated With Mental Health Disorders Among University Students in France Confined During the COVID-19 Pandemic	France	69 054 students	Students who completed it in its entirety the questionnaire 7114 respondents (10.3%) reported a history of psychiatric follow-up	Mean age of the sample: 20 years; 50 251 Female (72.8%)	To measure the prevalence of self-reported mental health symptoms, to identify associated factors, and to assess care seeking among university students who experienced the COVID-19 quarantine in France	Self-reported suicidal thoughts assessed using the 22-item Impact of Events Scale–Revised	Cross‐sectional survey	From April 17 to May 4, 2020	Among risk factors identified, reporting at least 1 outcome, including suicidal thoughts, was associated with history of psychiatric follow-up
*Every-Palmer* et al*. 2020*	Psychological distress, anxiety, family violence, suicidality, and wellbeing in New Zealand during the COVID-19 lockdown: A cross-sectional study	New Zealand	2416 subjects, cleaned achieved sample of 2010 cases	Representative sample of adult New Zealanders aged between 18 and 90 years recruited from a commercial survey platform 375 people (18.2%) reported previously been diagnosed with a mental health condition by a doctor or psychologist Of these, many had more than one diagnosis, with 80.2% reporting having been diagnosed with a depressive disorder, 52.6% anxiety disorder, 5.8% personality disorder, 7.6% bipolar disorder, 5.7% an alcohol and drug disorder, 3. 9% a psychotic disorder, and 11.4% another disorder	Mean age of the sample: 45 years; 1063 Female (52.9%)	To determine: - The state of the New Zealand population’s wellbeing during the COVID-19 lockdown (stress, anxiety, depressive symptoms, alcohol consumption, family relationships, suicidal thinking, etc.) - How the lockdown affected specific populations (e.g., essential workers, those with underlying health conditions, and the elderly) - Whether there were any positive psychological consequences associated with the lockdown	Suicidality assessed using questions on suicidal ideation, suicide plans, and suicide attempts during the lockdown and the preceding 12 months	Cross‐sectional survey	Between 15 and 18 April 2020	Over half of those with past mental health diagnoses were experiencing moderate or severe psychological distress. About one-third thought their mental health had been worse than usual during the lockdown (52.9%), just under half thought it was the same as usual (46.1%), and about one in six reported it was better than usual (17.5%). Suicidal ideation during lockdown reported by 6.1% of participants with 2.1% reporting making plans for suicide and 2.1% also reporting a suicide attempt For most of those experiencing suicidal thoughts, these were not new thoughts– 83.0% of that group reported having experienced similar ideation in the 12 months prior to lockdown Vulnerable groups included those with past history of mental illness
*Daly *et al*. 2021*	Associations between periods of COVID-19 quarantine and mental health in Canada	Canada	3558 subjects	Individuals from the Maru Voice Canada panel and who completed the survey 18.2% (546) reported having a pre-existing mental health condition	Age range of the sample: 18–34: 534, (17.8%); 35–54: 1157 (38.6%); 55 + : 1309 (43.6%); 1519 woman (50.6%)	To examine the relationships between COVID-19 quarantine and mental health, including suicidal ideation, and self-harm; and to explore whether mental health outcomes differ depending on the specific reason for quarantine	Suicidal ideation and self-harm were assessed by asking participants, “as a result of the COVID-19 pandemic, in the previous two weeks” had they “Experienced suicidal thoughts/feelings?” or “Deliberately hurt myself?	Cross‐sectional survey	between May 14–29, 2020	COVID-19 quarantine for any reason was associated with an increase in the odds of suicidal ideation in the group having a pre-existing mental health condition
*Bruffaerts* et al*. 2021*	Suicidality among healthcare professionals during the first COVID-19 wave	Belgium	6,409 healthcare professionals	Healthcare professionals. Lifetime problems with anxiety/nerves endorsed by 12.1%, then depression (7.7%), panic attacks (2.8%), and substance use problems (0.9%). Any lifetime problem estimated at 19.1%	Mean age of the sample: 41.6 years; 72.4% female	To investigate the 30-day prevalence of suicidal thoughts and behaviors (STB) and associated risk factors among clinically active healthcare professionals during the first wave of COVID-19 pandemic	A modified version of the Columbia Suicidal Severity Rating Scale used to assess STB, including suicidal ideation. Any STB defined as any positive answer on at least one of the STB questions	Cross‐sectional survey	Between 6 April and 14 July 2020	Prevalence of suicidal thoughts and behaviors (STB) was 3.6% death wish, 1.5% suicide ideation, 1.0% suicide plan, and 0.0% suicide attempt. Thirty-day suicidal thoughts and behaviors (STB) increased among respondents with lifetime mental disorders (mostly depression)
*Behera et* *al. 2021*	Trends in deaths attributable to suicide during COVID-19 pandemic and its association with alcohol use and mental disorders: Findings from autopsies conducted in two districts of India	New Delhi, India	321 between March–October 2020 331 between March–October 2019	Autopsies of deaths attributable to suicide from two districts in New Delhi	Mean age of the sample: 28.00 years; majority being male (235/321, 73.2%)	To assess the impact of situation consequent to COVID-19 pandemic on the deaths due to suicide in two districts of New Delhi, India	Psychological autopsy of deaths due to suicides examined to explore factors associated with suicide	Retrospective, Cross-sectional study	Pre-lockdown from 25 March 2019 to 31 October 2019 and during lockdown from 25 March 2020 to 31 October 2020	There was a significant decline in deaths due to suicide during the lockdown period. Psychological autopsy suggested mental disorders to be the underlying cause for suicidal behavior in 10.2% (33 out of 321) cases. There was a significantly greater proportion of deaths due to suicide attributable to mental illness (12.3% vs. 1.6%, p = 0.01) during the unlock period. There was a significantly lesser number of deaths due to suicide with past history of suicide attempt in the current as compared to the last year
*Shi *et al*. 2021*	Prevalence and correlates of suicidal ideation among the general population in China during the COVID-19 pandemic	China	56,679 subjects	Nationwide sample from 34 China province-level regions From the entire sample 161(0.3%) had a history of mental disorder In this subgroup 67 (0.7%) reported suicidal ideation	Age range of the sample: 18–24: 3,267 (5.8%), 25–34: 23,050 (40.7%), 35–44: 21,658 (38.2%), ≥ 45: 8,704 (15.4%); 27,149 (47.9%) males and 29,530 (52.1%) females	To explore the prevalence of suicidal ideation and its risk factors among the general population in China during the COVID-19 pandemic and further provide evidence for suicide prevention under a public health emergency	Suicidal ideation assessed according to the suicidal item of Patient Health Questionnaire, PHQ-9	Cross-sectional survey	Between February 28, 2020 and March 11, 2020	Suicidal ideation was more prevalent in individuals with pre-existing mental disorders (41.6%). Among factors associated with suicidal ideation during the COVID-19 pandemic, a history of mental disorders increased risk of suicidal ideation
*Yang *et al*. 2021*	The Differential Effects of Social Media on Depressive Symptoms and Suicidal Ideation Among the Younger and Older Adult Population in Hong Kong During the COVID-19 Pandemic: Population-Based Cross-sectional Survey Study	Chinese University of Hong Kong, Honk Kong	1070 subjects	Population-based sample. 25 (2.3%) were diagnosed with mental health problems before the pandemic; 20 (1.9%) were diagnosed with mental health problems during the pandemic	Age range of the sample: 18–35: 115 (10.7%); 36–55: 252 (23.6%); 56–65: 301 (28.1%); > 65: 383 (35.8%); Male 346 (32.3%); Female 724 (67.7)	To test the mediation effects of social loneliness and post-traumatic stress disorder (PTSD) symptoms on the relationship between social media use and depressive symptoms and suicidal ideation, as well as the moderation effect of age on the mediation models	Suicidal ideation assessed according to the suicidal item of Patient Health Questionnaire, PHQ-9	Cross-sectional survey	Between May 14 and June 4, 2020	Being diagnosed with mental health problems before or during the COVID-19 pandemic were positively associated with suicidal ideation
*Al-Humadi *et al*. 2021*	Depression, Suicidal Thoughts, and Burnout Among Physicians During the COVID-19 Pandemic: a Survey-Based Cross-Sectional Study	New York, USA	225 physicians	The sample was composed of residents, fellows, and attending physicians from 26 specialties working at Stony Brook University Hospital. 65 participants (29%) previously been diagnosed or treated for depression or anxiety	Mean age of the sample: 38.57 years; 129 female (57.3%)	The study investigates the incidence and associated factors of depression, suicidal thoughts, and burnout among physicians during the COVID-19 pandemic	Suicidal ideation assessed according to the suicidal item of Patient Health Questionnaire, PHQ-9	Cross-sectional survey	From April 4 through May 1, 2020	Suicidal ideation was associated with history of depression/anxiety, during the COVID-19 pandemic The prominence of premorbid depression/anxiety as a relevant factor underscores the need to further understand physician mental health and provide early screening and treatment
*Sáiz *et al*. 2020*	Prevalence of Passive Suicidal Ideation in the Early Stage of the Coronavirus Disease 2019 (COVID-19) Pandemic and Lockdown in a Large Spanish Sample	Spain	21,207 participants	Large general population sample aged 18 years or over 3,665 (17,3%) suffered a past mental disorder, 2489 (11,7%) suffered currently a mental disorder	Mean age of the sample: 39.7 years; 14,768 females (69.6%)	To determine the prevalence of passive suicidal ideation in a sample of the general Spanish population early in the COVID-19 pandemic and lockdown and to characterize factors associated with such thoughts	To determine independent factors associated with passive suicidal ideation subjects involved asked if they had: “passive suicidal ideation during past 7 days” (no/yes)	Cross-sectional survey	Between March 19 and 26, 2020	1,873 responders (8.8%) had experienced passive suicidal ideation during the past 7 days. Risk factors for passive suicidal ideation were having a personal history of past/current mental disorder
*Mamun *et al*. 2020*	Indian celebrity suicides before and during the COVID-19 pandemic and their associated risk factors: Evidence from media reports	India	23 press reports	Media reports The most common cause of reported suicide during the different time periods was depression (17) although family problems (3), bipolar disorder (1), and personal reasons (1) along with one case wherein the cause of suicide was unreported	Mean age of the sample: 37.94 years between 2002–2019, 26.43 years during the first three months of Covid-19 pandemic; 10 males and 6 females between 2002–2019, 3 males and 4 females during the first three months of Covid-19 pandemic	To assess suicide victims based on a specific high-profile occupation (celebrities working in the entertainment industry) and to examine the probable causality of suicides both before and during the COVID-19 pandemic period	Google News search engine used to retrieve relevant articles. The search terms included, ‘Indian cinema celebrities’, ‘celebrity suicides’, ‘COVID-19 pandemic’, ‘depression’, ‘financial strife’, and ‘lockdown related restriction’	Retrospective, Cross-sectional study	Between 2002–2019 and during the first three months of Covid-19 pandemic	While the 16 celebrity suicides prior to the COVID-19 pandemic spread over 17 years (2002–2019), the seven celebrity suicides during the COVID-19 pandemic occurred within a 3-month period. Depression was reported to be the most common cause of celebrity suicides both before and during the COVID-19 pandemic, but the increased incidence of celebrity suicides during the ongoing pandemic suggests a possible association between COVID-19-related restrictions and the exacerbation of pre-existing mental health conditions such as depression that increases the risk of suicidality among Indian celebrities
*Czeisler *et al*. 2020*	Mental Health, Substance Use, and Suicidal Ideation During the COVID-19 Pandemic — United States, June 24–30, 2020	Monash University, Melbourne, Australia, Harvard Medical School Boston, Massachusetts, USA	5,470 subjects	Adults who completed web-based surveys. Among subjects receiving treatment for previously diagnosed condition: 536 have Anxiety (9.8%), 540 Depression (9.9%), 251 Posttraumatic stress disorder (4.6%)	Age range of the sample: 18–24: 731 (13.4%), 25–44: 1,911 (34.9%), 45–64: 1,895 (34.6%), ≥ 65: 933 (17.1%); 2,784 Female (50.9%)	To assess mental health, substance use, and suicidal ideation during the pandemic	Respondents also reported whether they had seriously considered suicide in the 30 days preceding the survey	Cross-sectional survey	During April–June of 2020, compared with the same period in 2019	Overall, 40.9% of 5,470 respondents who completed surveys during June reported an adverse mental or behavioral health condition, including having seriously considered suicide in the preceding 30 days (10.7%). Suicidal ideation was also elevated; approximately twice as many respondents reported serious consideration of suicide in the previous 30 days than did adults in the United States in 2018, referring to the previous 12 months. Mental health conditions are disproportionately affecting specific populations including those receiving treatment for pre-existing psychiatric conditions
*Iob *et al*. 2020*	Abuse, self-harm and suicidal ideation in the UK during the COVID-19 pandemic	London United Kingdom	44 775 subjects	Data from University College London's (UCL's) COVID-19 Social Study; 8757 received a Mental health diagnosis (19.6%)	Age range of the sample: between 18–29 years: 7835 (17.5%), between 30–44 years: 10 394 (23.2%), between 45–59 years: 12 031 (26.9%), Over 60 years: 14 515 (32.4%); Female 22 846 (51.0%)	To address evidence gaps by exploring patterns of abuse, self-harm and thoughts of suicide or self-harm in the UK in the first month of lockdown due the COVID-19 pandemic and exploring whether those having such experiences were accessing formal or informal mental health support	Suicidal ideation assessed according to the suicidal item of Patient Health Questionnaire, PHQ-9	Cross‐sectional survey	Between 21 March and 20 April 2020	7984 participants (18%) reported experiencing thoughts of suicide or self-harm in the first month of lockdown and 2174 participants (5%) reported harming themselves at least once since the start of the UK's lockdown. In the first month of lockdown in the group of subjects with a mental health diagnosis 3813 reported self-harm/suicidal thoughts 3813 (43.5%); 1241 reported self-harm behaviors (14.2%). The patterning of thoughts and experiences of self-harm during the first month of lockdown included having a mental disorder
*Panigrahi *et al*. 2021*	COVID-19 and suicides in India: A pilot study of reports in the media and scientific literature	India	151 reports	COVID-19 related suicides (CRS) reports 7 had a pre-existing psychiatric illness, depression	Mean age of the sample: 38.7 years; 19.2% (29) were females	To analyze reports of COVID-19 related suicides (CRS) to identify associated factors with a broader goal to inform management and prevention strategies	Search scientific literature, government websites and online newspaper reports in English and nine regional languages to identify relevant COVID-19 related suicides (CRS) reports	Retrospective, Cross-sectional study	Between 1st February 2020 to 30th September 2020	The study suggests that socio-demographic factors, stigma related to a diagnosis of COVID-19, being in quarantine/isolation and recent physician contact are markers of COVID-19 related suicides CRS Among the deceased, majority (89.4%, 135), had no comorbid physical/mental illness or substance use
*Kasal *et al*. 2022*	Suicide Risk in Individuals With and Without Mental Disorders Before and During the COVID-19 Pandemic: An Analysis of Three Nationwide Cross-Sectional Surveys in Czechia	Czech Republic	May 2020 dataset consisted of 3,021 participants, 2017 dataset consisted of 3,306 respondents, November 2020 dataset consisted of 3,000 participants	Data from three nationally representative cross-sectional surveys of Czech community-dwelling adults Subjects with a major depressive episode were: 132 (3.99%) in 2017 May sample, 359 (11.88%) in 2020 May sample, 382 (12.73%) in 2020 November sample; Anxiety disorders were: 261 (7.89%) in 2017 May sample, 408 (13.51%) in 2020 May sample, 398 (13.27%) in 2020 November sample	Mean age of the sample: 48 years 2017 May sample, 46 years 2020 May Sample, 46 years 2020 November Sample; 2017 May sample: 1,774 females (53.66%), 2020 May sample: 1,581 females (52.33%), 2020 November Sample: 1,534 females (51.13%)	To assess changes in suicide risk (SR) in people with and without mental disorders, before and during the COVID-19 pandemic in Czechia	Past month SR was assessed using a separate M.I.N.I. module consisting of 6 questions (1) “Think that you would be better off dead or wish you were dead?”, (2) “Want to harm yourself?”, (3) “Think about suicide?”, (4) “Have a suicide plan?”, and (5) “Attempt suicide?”, (6) “Did you ever make a suicide attempt?”. In line with the scoring procedure proposed by the authors of the instrument, we considered a positive answer to any question as indicative of past-month SR. A positive answer to any of the first four and last two items was indicative of presence of ST and suicidal behavior (SB), respectively	Analysis of Three Nationwide Cross-Sectional Surveys, paper and pencil interviewing, while for the two 2020 data collections, we used a mixed computer-assisted web interviewing and computer-assisted telephone interviewing approach	Three nationwide cross-sectional surveys: November 2017, May and November 2020	Individuals with anxiety disorders exhibited an increase of approximately 12% and 20% in SB prevalence compared to the baseline, major depressive disorder was associated with higher odds of SR in all three datasets
*Mortier et. al 2021*	Thirty‐day suicidal thoughts and behaviors among hospital workers during the first wave of the Spain COVID‐19 outbreak	Spain	5450 hospital workers	Cohort of Spanish hospital workers 568 had a lifetime mood disorder, 1893 had a lifetime anxiety disorder	Mean age of the sample: 42.9 years in those that completed the STB items, 42.1 years in those that did not; females 80.8% in those that completed the STB items, females 82.1% in those that did not	To examine baseline prevalence of 30‐day STB and to investigate the relationship of potentially modifiable contextual factors related to hospital workers' perceived work and financial situation, with 30‐day STB	Modified version of selected items from the Columbia Suicide Severity Rating Scale	Cross-sectional survey	From May 5–July 23, 2020	Thirty‐day suicidal thoughts and behaviors (STB) prevalence was estimated at 8.4% 6 professionals attempted suicide in the past 30 days. In adjusted models, 30‐day suicidal thoughts and behaviors (STB) remained significantly associated with pre‐pandemic lifetime mood and anxiety disorder

**Table 5:** Suicide and Covid-19 in subjects with pre-existing mental disorder, other studies

*Characteristics of other studies examining Covid-19 and suicide in subjects with pre‐existing mental health conditions (n* = *11)*										
*Authors,* *year*	Title	Location of study	Sample size	Nature of sample	Age range of the sample and gender	Purpose/aim of the study	Suicide assessment measures	Type of publishing	Time points compared/analyzed in the study	Principal findings
*Hedley et* *al. 2021*	The association between COVID-19, personal wellbeing, depression, and suicide risk factors in Australian autistic adults	La Trobe University, Melbourne, Australia	111 Autistic patients	Autistic adults aged 20 to 71 years during the second wave of the COVID-19 pandemic in Australia Co‐occurring diagnoses of anxiety or depression were reported by 70% (73, 71, respectively) of participants	Mean age of the sample: 42.45 years; 58,6% women and 32,4% men	To examine potential associations between COVID-19 impact and depression, personal wellbeing, and suicide risk factors in Australian autistic adults and age and gender effects	Suicide risk assessed using Suicide Behavior Questionnaire Revised (SBQ-R)	Mixed‐method survey design	Between October and December 2020	The impact of the COVID-19 pandemic may be associated with poorer wellbeing and higher depression but is not associated with suicide risk in this autistic adults’ sample
*Carlin et. al 2021*	Impact of COVID-19 lockdown on suicide attempts A retrospective analysis of the springtime admissions to the trauma resuscitation room at the Medical University of Vienna from 2015–2020	Trauma Centre of the Medical University of Vienna, Vienna, Austria	559 admissions; 79 in 2015; 87 in 2016; 110 in 2017; 109 in 2018; 109 in 2019; 65 in 2020	Patients admitted to the trauma resuscitation room. Number of patients in the control group was 37, in the study group 23. Patients with known psychiatric disease were 32 (86.5%) in the control group and 19 (82.6%) in the study group. Patients with history of previous suicide attempt were 13 (35.1%) in the control group and 9 (39.1%) in the study group	Mean age of the sample: 43,2 years in the control group; 38.7 years in the study group; majority being male in both groups	To analyze how many of the patients admitted to the trauma resuscitation room of the level 1 Trauma Centre of the Medical University of Vienna during the COVID-19 lockdown in Austria injured due to a suicide attempt by intentionally caused trauma	Retrospective data analysis. Patients subdivided into trauma victims and individuals who had attempted suicide	Mixed‐method, Case–control, Cross-sectional study	From 16 March to 15 May in the years 2015–2019	The study revealed a significantly higher proportion of attempted suicides in all patients admitted to the trauma resuscitation room Due to the small number of patients in the study group an increase in suicidal behavior isolated for individuals with previous mental health problems could not be observed, but more generally an increase in suicidality across different groups at risk
*Czeisler *et al*. 2021*	Mental health, substance use, and suicidal ideation during a prolonged COVID-19-related lockdown in a region with low SARS-CoV-2 prevalence	Victoria region, Australia	1531 subjects	Victorians who completed the surveys History of diagnosed psychiatric condition was present in: 123 subjects (37.1%) in the Victorian-April group; 435 subjects (37.6%) in the Victorian-September group; 38 subjects (41,4%) in the Victorian-Longitudinal group	Age range of the sample: Age range in the Victorian-April group: 18–24: 42 (12.8%); 25–44: 123 (10.6%); 45–64: 105 (31.7%); ≥ 65: 61 (18,4%); Age range in the Victorian-September group: 18–24: 123 (10.6%); 25–44: 436 (37.6%); 45–64: 379 (32.8%); ≥ 65: 219 (18.9%); Age range in the Victorian-Longitudinal group: 18–24: 11 (12.3%); 25–44: 34 (36.5%); 45–64: 29 (31.1%); ≥ 65: 19 (20.2%)	To analyze the associations between adverse mental and behavioral health symptoms and demographic characteristics, sleep, and behavioral changes, with the aim of identifying areas for targeted interventions to improve mental health	Data collection including past-month passive suicidal ideation (i.e., wished to be dead), and past-month serious suicidal ideation	Mixed‐method Longitudinal, cross-sectional survey	During April 2–8, 2020 (April-2020) and September 15–24, 2020	Suicidal ideation was nearly three times as prevalent among respondents with *vs* without previously diagnosed psychiatric conditions. Diagnosed psychiatric disorder was associated with poor outcomes, including suicidal ideation, during COVID-19 pandemic
*Hyland* et al*. 2021*	Predicting risk along the suicidality continuum: A longitudinal, nationally representative study of the Irish population during the COVID‐19 pandemic	Ireland	1,032 subjects 715 who completed the follow-up	Nationally representative sample of Irish adults Some participants reported a history of mental health treatment	Mean age of the sample: 44.86 years; 52.1% female	To analyze the lifetime prevalence of different indicators of suicidality in the Irish general population; whether suicidality has increased during the COVID‐19 pandemic; and what factors associated with belonging to different points on a continuum of suicidality risk	Three items were adapted from the 2014 English Adult Psychiatric Morbidity Survey to measure suicidal and self‐harm ideation	Mixed‐method Longitudinal, cross-sectional survey	May 2020 and a follow‐up in August 2020	There was no statistically significant change over time in suicidal behavior. Suicidal ideation was not associate with been treated for a mental health problem. Those who treated for a mental health problem were over twice as likely as those with no such history to have engaged in non‐suicidal self‐injury (NSSI). Among variables significantly associated with attempted suicide there was having received treatment for a mental health problem
*Veldhuis* et al*. 2021*	Addressing the critical need for long-term mental health data during the COVID-19 pandemic: Changes in mental health from April to September 2020	USA and more than 50 other countries	1567 subjects	Longitudinal data from an international convenience sample of adults 18 or older living in every US state and more than 50 countries who completed baseline surveys and the 5-month follow-up. 58.9% received a mental health diagnosis. Participants who reported depression, anxiety, or PTSD were considered to have a previous relevant mental health diagnosis	Age range of the sample: 18–30: 38.0%; 31–40: 31.8%; 41–50: 14.2%; 51–65: 12.3%; 66 + : 3.7%; Women 88.2%	To better understand the longer-term effects of the pandemic on mental health	Suicide risk was measured using the Suicidal Ideation Attributes Scale (SIDAS) which measures thoughts and behaviors and provides an assessment of risk (scores of 21 or higher are considered severe risk and 10 or higher is considered to be some risk)	Mixed‐method Longitudinal, cross-sectional survey	Baseline surveys during April 5–19, 2020; 5-month follow-up (August 28–September 11, 2020	Baseline risk for suicide was associated with 12 times higher odds of risk for suicide at 5-month follow-up. Having received a mental health diagnosis was associated with suicide risk at 5-month follow-up
*Hamm* et al*. 2020*	Experiences of American Older Adults with Pre-existing Depression During the Beginnings of the COVID-19 Pandemic: A Multicity, Mixed-Methods Study	Los Angeles, New York, Pittsburgh, and St Louis, USA	73 older adults with pre-existing Major depressive disorder	Community-living older adults with pre-existing Major depressive disorder (MDD) recruited among the 743 participants in the Optimizing Outcomes of Treatment-Resistant Depression in Older Adults (OPTIMUM) clinical trial	Mean age of the sample: 69 years; 50 females (68.5%)	To determine the effect of the COVID-19 pandemic on the mental health of older adults with pre-existing major depressive disorder (MDD)	Suicidal ideation assessed according to the suicidal item of Patient Health Questionnaire, PHQ-9	Multicity, Mixed-Methods Study	Between April 1 and April 23, 2020	Examination of PHQ-9 item 9 (thoughts of death or self-harm) revealed no increase in suicidal thoughts. Of 72 with scores available in the immediate peripandemic period, 59 (82%) had no thoughts of death (score of 0) both beforehand and currently; 5 (7%) had current thoughts of death (all had score of 1) but none beforehand; while 7 (10%) had reduced thoughts of death currently compared to before the pandemic (score of 0 currently vs. 1 beforehand in 5 participants, score of 1 currently vs. 3 beforehand in 2); 1 refused to answer
*Knipe et* *al. 2022*	Hospital presentations for self-poisoning during COVID-19 in Sri Lanka: an interrupted time-series analysis	Teaching Hospital Peradeniya, Sri Lanka	1401 individuals; Pre-pandemic period (1161) Pandemic period (240)	Hospital admissions for self-poisoning. Subjects with a current psychiatric diagnosis were 535 in pre-pandemic period (46,1%) and 110 in pandemic period (45,8%)	Age range of the sample: Pre-pandemic period < 25 years were 603 (51,9%), Pre-pandemic period ≥ 25 years were 546 (47,0%); Pandemic period < 25 years were 107 (44,6%), Pandemic period ≥ 25 years 117 (48,8%); 761 (54,3%) females	To determine the effect of the pandemic on hospital presentations for self-poisoning	Admission book data and information from bed head tickets were used to identify cases of self-poisoning (intentional self-harm by ingesting poison). Medical records used to retrieve all relevant information regarding the patient's stay, including any related history. The overall number of admissions per month were collected from the admission books and used to trace bed head tickets for detailed information regarding admissions (type of poison ingested)	Interrupted time-series analysis	Before (Jan 1, 2019–March 19, 2020) and during (March 20–Aug 31, 2020) the pandemic	A sudden drop in presentations for self-poisoning occurred at the start of the lockdown period; on average, there were more presentations in the pre-pandemic period than in the pandemic period. The time-series analysis indicated that there was a 32% reduction in hospital presentations for self-poisoning during the pandemic period compared with the pre-pandemic period. There was no statistical evidence that the impact of the pandemic differed regarding having a current psychiatric diagnosis
*McDowell *et al*. 2021*	Evaluating the association between COVID-19 and psychiatric presentations, suicidal ideation in an emergency department	Massachusetts General Hospital, Boston, Massachusetts, United States	2018–2019 Cohort (489) 2019–2020 Cohort (467)	Psychiatric Presentation in an emergency department In the 2018–2019 Cohort, patients presenting with suicidal Ideation were 293 (59.9%), substance use disorder were 202 (41.3%), affective disorder were 172 (35.2%), psychotic disorder were 83 (17.0%); In the 2019–2020 Cohort, patients presenting with suicidal Ideation were 274 (58.7%); substance use disorder were 184 (39.4%); affective disorder were 184 (39.4%); psychotic disorder were 57 (12.2%)	Mean age of the sample: 38 years in the 2018–2019 Cohort, 39 years in the 2019–2020 Cohort; 281 female (57.5%) in the 2018–2019 Cohort; 301 female (58.0%) in the 2019–2020 Cohort	To estimate the association between COVID-19 and Emergency Department (ED) psychiatric presentations, including suicidal ideation	The presence or absence of suicidal ideation as determined by text in the psychiatric consultation note, as well as up to three psychiatric diagnoses (not including SUDs), recorded for each patient presentation	Interrupted time-series analysis	Between 2018 and 2020, February 26–March 6, 2020 used to define patterns in psychiatric presentations before and after the coronavirus outbreak	Results estimate significant differential change associated with suicidal ideation and substance use disorder (SUD) presentations following the outbreak. Specifically, there were a significant differential increase in presentations with suicidal ideation six weeks after the outbreak (36.4 percentage points change) For presentations with SUD, there were a differential increase in the COVID-19 time series relative to the comparison time series at all post-outbreak time points and this differential increase was significant three weeks (32.8 percentage points; 95% CI: 4.0, 61.6) following the outbreak. Moreover, SUD presentations seem to explain the increase in suicidal ideation presentations in week 3 in the unadjusted models. The results estimate no differential changes significant at the P value < 0.05 level associated with affective disorder or psychotic disorder presentations in the COVID-19 time series relative to the comparator time series
*Oliè *et al*. 2021*	Psychological state of a sample of patients with mood disorders during the first French COVID-19 lockdown	France	69 healthy controls (HC) 346 patients with a major depressive episode in the two previous years (PP)	Patients with history of depressive episodes and healthy controls. In the PP group: 44.5% (50) had a diagnosis of bipolar disorder, 50% (174) lifetime history of suicide attempt, 62% (207) lifetime history of anxious disorder, 22.2% (74) alcohol abuse or dependence, 15.2% (51) illicit substance abuse or dependence, and 15.2% ( 51) an eating disorder	Mean age of the sample: healthy controls (HC) 37 years, patients with a major depressive episode in the two previous years (PP) 39 years; 16 men (23.2%) healthy controls(HC) ,83 men (24%) patients with a major depressive episode in the two previous years (PP)	To compare in 69 healthy controls (HC) and 346 patients with a major depressive episode in the two previous years (PP) self-reported psychological symptoms (depression, anxiety, insomnia, suicidal ideation, traumatic stress, anger) and living conditions during the first national French lockdown, and identify predictors of significant psychological distress in PP	Suicidal ideation assessed according to the suicidal item of Patient Health Questionnaire, PHQ-9	Case–control study	From March, 17 to May 11, 2020	Current suicidal ideation (according to the suicidal item of Patient Health Questionnaire, PHQ-9) was reported by 26.6% of PP (92). Suicidal ideation was predicted by psychotropic drug changes, history of suicide attempt, high education level, and loneliness. Daily virtual contacts were protective against suicidal ideation
*Louie *et al*. 2021*	Suicidal Risk in Older Patients with Depression During COVID-19 Pandemic: a Case–Control Study	The University of Hong Kong, Queen Mary Hospital, Hong Kong	64; 31 healthy older adults, 33 adults with late-life depression (LLD)	Adults diagnosed with major depressive disorder (single or recurrent episode) as defined by the DSM-5 recruited from psychiatric clinics or inpatient wards, whereas healthy older adults without a history of depression or other psychiatric illnesses were recruited from voluntary organizations or elderly community centers	Mean age of the sample: 74.45 years adults with late-life depression (LLD), 71.10 years controls; 21 men and 43 women aged 61 to 89 years	To compare older adults with late-life depression (LLD) and healthy controls in terms of suicidal ideation during the COVID-19 pandemic, and to determine predictors of suicidal ideation	Suicide ideation assessed using Geriatric Suicide Ideation Scale (GSIS)	Case control study	Between March and April 2020	The level of suicidal ideation was significantly higher in the LLD group than the healthy control group after adjusting for depressive symptoms. Older people with LLD had a significantly higher suicidal risk during the COVID- 19 pandemic
*Hao *et al*. 2020*	Do psychiatric patients experience more psychiatric symptoms during COVID-19 pandemic and lockdown? A case–control study with service and research implications for immunopsychiatry	Chongqing, China	76 psychiatric patients 109 healthy control	An online questionnaire was administered via SMS to psychiatric patients from the databases of the First People’s Hospital of Chongqing Liang Jiang New Area, China The healthy control participants were recruited through convenient sampling For psychiatric patients, majority of the respondents had mixed anxiety and depressive disorder (59%), followed by other anxiety disorders (25%) and major depressive disorder (16%)	Mean age of the sample: 32.8 years Psychiatric patients, 33.1 years Healthy controls; Psychiatric patients 51 Female (37.1%), Healthy controls 68 Female (62.4%)	To assess and compare the immediate stress and psychological impact experienced by people with and without psychiatric illnesses during the peak of 2019 coronavirus disease (COVID-19) epidemic with strict lockdown measures	Structured questionnaire consisted of questions in which Suicide ideation was evaluated trough: 6- Other psychiatric symptoms	Case–control study	The psychiatric patients were recruited from 19 to 21 February 2020, and healthy control participants were recruited from 21 to 22 February 2020	Nine (11.8%) psychiatric patients and 1 (0.9%) healthy control reported moderate to severe suicidal ideation Specifically, in the Psychiatric patients (76) group: 64 (84.2%) reported No suicidal ideation, 3 (3.9%) Mild suicidal ideation, 5 (6.6%) Moderate suicidal ideation, 3 (3.9%) Serious suicidal ideation, 1 (1.3%) Very serious suicidal ideation

**Fig.1:**
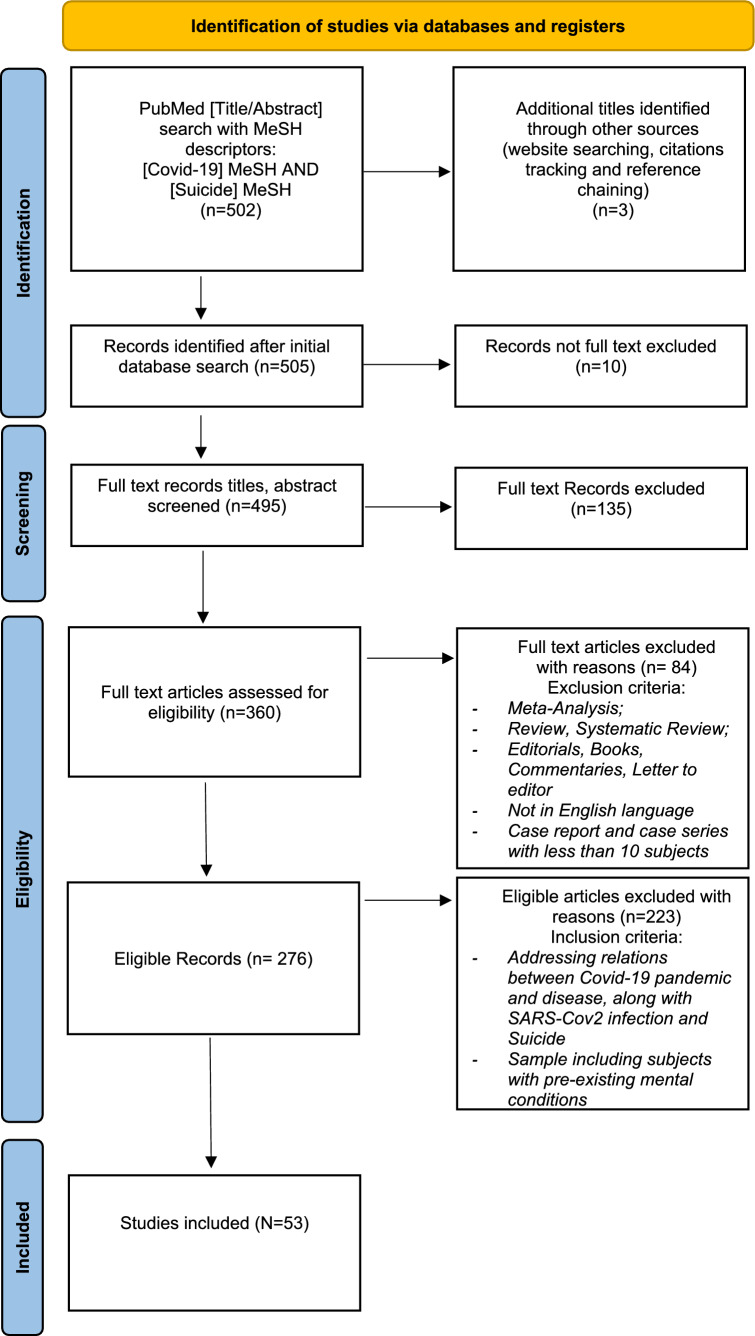
PRISMA 2020 flow diagram of included studies

For specifics about the study design, consult Fig. 1.

## Narrative overview

Among the 53 articles identified as eligible for directly assessing modifications of suicidal behavior during COVID-19 pandemic in patients with pre-existing mental disorders, 33 are cross-sectional studies, 9 are longitudinal studies, and 11 studies employed other methods.

Four longitudinal studies assessed in this review included patient samples, and the remaining 5 studied this correlation in general population samples.

Focusing on longitudinal investigations in patient samples, the 4 articles were carried out in different countries, namely 1 in Iran, 1 in Spain, 1 in the USA, and 1 in Denmark [19–22]. Two studies assessed suicide behavior in patients previously diagnosed with obsessive–compulsive disorder (OCD) [19, 20], one in a nationally representative cohort of US veterans with pre-existing mental health conditions [21] and one in individuals admitted to hospitals and Emergency Medical Services presenting psychopathological symptomatology [22]. In the study conducted in the USA by Na et al., suicidal behavior was assessed by means of the Patient Health Questionnaire-9 (PHQ-9); the two studies, carried out in Spain by Alonso et al. and in Iran by Khosravani et al., assessing patients with OCD employed Hamilton Depression Rating Scale (HDRS) item on suicide and Beck Scale for Suicidal Ideation (BSSI), respectively. The Danish study collected data regarding diagnoses from electronic health records (EHRs)—including codes for suicide and self‐harm—defined and coded according to the ICD‐10 system by the responsible clinicians. 3 out of 4 studies identified mental illness as a risk factor for suicide and addressed a relationship between the outbreak of the pandemic and an increase in suicidal behavior. Specifically, the study conducted by Na et al. relates ongoing SARS-CoV2 infection with an increased risk of suicidal behavior in subjects already suffering from a mental disorder. The study conducted in Denmark revealed how most patients exhibiting suicidal behavior during the pandemic presented a pre-existing mental disorder. However, the hospital‐registered rate of suicidal events during the pandemic significantly did not change when compared to the pre‐pandemic period. For specifics about these studies, see Table 1.

Among longitudinal studies using a general population sample, 1 was carried out in Greece, 2 in the USA, 1 in Australia, and 1 was an international study spanning over 40 countries and including up to 55,589 participants [23–27]. Two studies assessed suicidal behavior among US veterans [24, 25], and 2 studies analyzed national representative samples from Australia (by Batterham et al.) and Greece (by Fountoulakis et al.), respectively, evaluating suicide risk by means of the suicidal item of PHQ-9 and Risk Assessment Suicidality Scale (RASS); the two studies among veterans used Suicidal Behaviors Questionnaire-Revised (SBQ-R) and PHQ-9. The international study used the RASS for suicide assessment. Most of the studies demonstrated a correlation between the presence of suicidal behavior during the pandemic with previous mental health conditions. Conversely, the study carried out by Batterham et al. in Australia evidenced that a previously diagnosed psychiatric disorder, despite being a risk factor for suicidal behavior, is not significantly associated with incident suicidal ideation during the pandemic. The study conducted in Greece highlighted how a previous history of depression, self-harm, and suicidal attempts represent risk factors for relapsing depression and, eventually, suicidality during the pandemic. The study carried out by Nichter et al. underlined how a history of suicide attempt, lifetime post-traumatic stress disorder and/or depression, and past-year alcohol use disorder severity can be classified as risk factors, among COVID-19-related variables, for new-onset suicidal ideation. The international study conducted among 40 countries highlighted how suffering from a previous mental condition acted as a risk factor and suicidal behavior resulted increased in those people during pandemic. For specifics about these studies, see Table 2.

When screening cross-sectional studies, we identified 12 conducted on patient’s sample and 21 on general population samples.

Among cross-sectional studies analyzing the overstated correlation in patients with a pre-existing psychiatric disorder, one was carried out in China, 3 in Italy, 2 in South Korea, 2 in the USA, 1 in Germany, 1 in Turkey, 1 in Denmark, and 1 in Saudi Arabia [8, 28–38]. Most were service utilization studies, gaining clinical information from hospital admission records or clinical records. The study conducted in China by Liu et al. assessed suicide risk using 3 standardized ("yes" or "no") questions in older clinically stable patents with psychiatric disorders [28]. Two Italian studies were conducted by the same research group (from Sant'Andrea Hospital in Rome), one, by Montalbani et al., used Columbia Suicide Severity Rating Scale (C-SSRS) for suicide assessment, while the other, carried out by Berardelli et al. evaluated suicide attempt (SA) at the time of hospital admission, and suicide ideation (SI) and non-suicidal self-injury (NSSI) by means of the C-SSRS as well [29, 30]; in the study conducted by Almaghrebi et al. in Saudi Arabia suicide risk factors were assessed by means of the Modified SAD PERSONS Scale (MSPS) [8]. The two Korean studies recorded data on patients and assessed suicide lethality with the Risk-Rescue Rating in Suicide assessment (RRRS) and the severity of the suicide attempt on the South Korean Triage and Acuity Scale (KTAS) [31, 32]. The Turkish study focused on relapse rates defining criteria, including new-onset suicide behavior or ideation, to assess suicide risk during the first trimester since the declaration of the pandemic [33]. In the study conducted in Germany, Seifert et al. performed Psychopathological Assessment (PPA) according to the "Arbeitsgemeinschaft für Methodik und Dokumentation in der Psychiatrie" (AMDP)-System on patients presenting to the psychiatric emergency department [34]. Grossman et al., in Massachusetts (USA), obtained data from notes in clinical records regarding suicidality in patients presenting in the emergency department [35]. A study conducted in Kaiser Permanente hospital in Northern California by Ridout et al. assessed population-level incidence rate ratios (IRRs) and percent relative effects for suicide-related emergency department encounters [36]. Jefsen et al., in the Danish study, categorized clinical notes according to diagnosis and identified five distinct categories according to different clinical presentations of suicidal behavior: 1—thoughts of self‐harm, 2—completed self‐harm, 3—passive wish to die of COVID-19, 4—suicidal thoughts, 5—suicide attempts [37]. Slightly more than half of the considered studies evidenced an increase in suicidal behavior, hospital consults, and admissions among patients during the pandemic, underlying the role of pre-existing mental conditions as a risk factor for suicidal behavior. Interestingly, in the study conducted by Liu et al., among the patients exhibiting suicidal behavior, Major Depressive Disorder (MDD) was found to be the most common psychiatric pre-existing diagnosis [28]. Conversely, in the study conducted by Menculini et al. in Perugia (Italy), more than one-third of the considered sample (patients presenting to the emergency room requiring psychiatric consultation) did not report any previous psychiatric history. Authors suggest that a percentage of cases were to be considered as new-onset suicidality, contrasting previously reported findings in which suicide and suicidal behavior were mostly related to pre-existing severe psychiatric disorders [38]. The study conducted by Lee et al. showed how the history of previous suicide attempts and previous psychiatric history were not significant independent risk factors for low-rescue suicide attempts when compared to COVID-19 as a risk factor itself [32]. The study conducted in Germany showed that the rate of patients self-reporting suicidal ideation and intent remained stable between 2019 and 2020. Suicidal ideation was stated significantly more often by patients with substance use disorders in 2020 than in 2019 [34]. Grossman et al. highlighted how accesses to psychiatric care in the COVID-19 post-period groups (case and comparator) were less likely to ascribe suicidality to psychiatric symptoms compared to visits in the comparator post-period group [35]. Finally, the Turkish study underlined how the relapse rate of the sample in 2019 did not differ from the first trimester of COVID-19 [33]. For specifics about these studies, see Table 3.

Among cross-sectional studies conducted on general population samples, 4 were carried out in Spain, 1 in Japan, 1 in Argentina, 1 in Latvia, 1 in Greece, 1 in France, 1 in New Zealand, 1 in Canada, 1 in Belgium, 3 in India, 1 in China, 1 in Honk Kong, 1 in the USA, 1 among Australia and USA, 1 in the UK, 1 in the Czech Republic [39–50]. Many of these studies assessed suicide behavior among a nationally representative sample; three of those focused on suicide among hospital workers [39–41] two studies analyzed COVID-19-related suicide reported by media, respectively [42, 43] and one assessed suicidal thought through the use of the 22-item Impact of Events Scale-Revised (IES-R) among 69.054 students in France [44], while the study carried out by Behera et al. analyzed autopsies of deaths attributable to suicide [45]. The studies conducted in Spain approached this correlation by employing different scales to assess suicide rates [39, 46–48]. Two studies used selected items from a modified version of the C-SSRS [39, 46], while two other Spanish studies, both conducted by Sàiz et al., investigated suicide behavior by means of the Paykel Suicide Scale (PSS) and by questioning participants on whether they experienced "passive suicidal ideation during the past seven days", requiring only yes/, no answers [47, 48]. The study conducted in Belgium used a modified version of selected items from the C-SSRS as well [49], while the Argentinian group employed the Inventory of Suicide Orientation (ISO-30) [50]. Finally, in Latvia, the authors assessed suicidal behavior with RASS [51].

Several different studies explored suicidal ideation by means of the suicidal item of the PHQ-9 [40, 42, 43, 52–59]. Some studies also evaluated suicide behavior without recurring to a validated questionnaire: Kasal et al. assessed the past month's suicide risk using a separate MINI module consisting of 6 questions [55]; in Japan, suicidal ideation was measured through a one-item question with different answer options [56]; in the New Zealand study, participants were interrogated on suicidal ideation, suicide plans, and suicide attempts during the lockdown and the preceding 12 months [57]. Daly et al. too assessed suicidality during the COVID-19 pandemic by directly questioning the participants whether they had ever experienced suicidal thoughts and feelings or past episodes of NSSI [58]. In a study conducted by Harvard University (Boston, Massachusetts, USA) and Monash University (Melbourne, Australia), respondents were required to report whether they had seriously considered suicide in the 30 days preceding the survey [59]. Two Indian studies searched scientific literature, government websites, an online newspaper, and google news to obtain information about suicides during the pandemic [42, 43]. Almost all the studies evidenced an increase in suicidal behavior, mostly related to a pre-existing mental health condition. In the study carried out by Behera et al., the authors suggest that the recurrence of psychiatric symptoms in individuals already diagnosed with mental conditions, such as depression, was associable with an increase in the risk of suicidality among Indian celebrities during the pandemic [45]. Also, Kasal et al. associated Major Depressive Disorder with a higher probability of suicide risk in three different datasets [55]. Al-Humadi et al. study concluded that suicidal ideation was almost entirely associated with a history of depression/anxiety during the COVID-19 pandemic [40]. Moreover, Vrublevska et al. observed an increase in suicidal thoughts of about 13.30% in participants with a history of clinically diagnosed depression and 27.05% in those with a history of suicide attempts during a state of emergency [51]. Conversely, Panigrahi et al. observed that the majority of those deceased by suicide (89.4%, 135) had no comorbid physical/mental illness or substance use [43]. For specifics about these studies, see Table 4.

Finally, we included 11 studies with different methodologies examining the relationship between the COVID-19 pandemic advent and suicide in subjects with pre-existing mental health conditions. Among the selected articles, three of them are case–control studies, respectively, conducted in France, China, and Hong Kong [36, 60, 61]; six are mixed-method studies, one carried out in Austria, two in Australia, one in Ireland, and two in the United States [59, 62–66]; finally, two interrupted time series analysis, from the USA and Sri Lanka, were included [67, 68]. Hedley et al. conducted one of the two studies from Australia and analyzed the impact of the COVID-19 pandemic on a sample of adults diagnosed with Autism Spectrum Disorders by means of a mixed‐method survey design [63]. Veldhuis et al., 2021, conducted a longitudinal survey in 2021 in the USA within a cross-sectional baseline assessment to obtain a better understanding of the long-term effects of the pandemic on mental health [59]. The second study conducted in Australia's Victoria region by Czeisler et al. utilized mixed methods as well, a cross-sectional survey and a longitudinal follow-up [64]. In Ireland, Hyland et al. carried out a cross-sectional analysis in May 2020 on a nationally representative sample of Irish adults (including 1,032 subjects), followed by a longitudinal reassessment carried out in August 2020 [65]. Aiming to estimate the association between the COVID-19 pandemic and Emergency Department (ED) psychiatric presentations (that included suicidal ideation), McDowell et al., from Massachusetts General Hospital in Boston, analyzed the time frame between 2018 and 2020 by employing an interrupted time series analysis [66]. In Hong Kong, Louie et al. carried out a study between March and April 2020, where 33 old adults diagnosed with Major Depressive Disorder (single or recurrent episode, as defined by DSM-5 criteria) were recruited from psychiatric clinics or inpatient wards and eventually compared with 31 healthy older adults with no history of depression [61]. In Sri Lanka, Knipe et al. carried out an interrupted time-series analysis in order to determine the effect of the pandemic on hospital presentations, with a focus on self-poisoning [68]. Carlin et al. carried out a retrospective analysis in the Trauma Centre of the Medical University of Vienna to analyze whether the COVID-19 pandemic affected the rates of hospital admission of patients who attempted suicide by intentionally causing trauma [62]. A retrospective case–control study was also conducted in China, where Hao et al. assessed the immediate stress and psychological impact of the initial phases of the COVID-19 pandemic in 2019 and compared the results between healthy controls and individuals affected by psychiatric illnesses [60]. A case–control study conducted in France assessed, through a cross-sectional survey, both the presence of psychological symptoms and living conditions in two distinct groups, healthy controls (HC) and patients with a recent (within the last 2 years) major depressive episode (PP); results were eventually compared and predictors of significant psychological distress in the PP group were identified [36]. Hamm et al. evaluated through a multicity, mixed-methods (both quantitative and qualitative) study the effect of the COVID-19 pandemic on 73 Community-living older adults with a pre-existing history of Major Depressive Disorder (MDD), aiming to explore this relationship in an older population with a previously diagnosed psychiatric condition [69]. In the study carried out by Veldhuis et al., suicide risk was assessed by means of the Suicidal Ideation Attributes Scale (SIDAS) administered to 1567 subjects from the USA to obtain measures of suicidal thoughts and behaviors and provide an assessment of overall risk [59]. In the study carried out in Hong Kong, suicidal ideation was assessed using Geriatric Suicide Ideation Scale (GSIS) in a sample of adults (healthy controls vs. patients with late-life depression diagnosis) [61]. Two separate studies by Hamm et al. and by Olié et al. assessed suicidal ideation by means of the suicidal item of the PHQ-9. [67] [44] Hyland et al. adapted three items from the 2014 English Adult Psychiatric Morbidity Survey to measure suicidal and self‐harm ideation [65]. On the other hand, Hao et al. assessed suicide ideation by means of a structured questionnaire [60]. In the Australian study conducted in the Victoria region, authors collected data that included both past-month passive suicidal ideation (i.e., wished to be dead) and past-month serious suicidal ideation [64]. Admission book data and information from bedhead tickets were used in the Sri Lanka study to identify self-poisoning cases [68]. McDowell et al. gained data about the eventual presence or absence of suicidal ideation by reading psychiatric consultation notes [66]. Almost half of the studies found no correlation between previous mental health diagnoses and suicide behavior rate changes during the pandemic. For instance, Knipe et al. observed a drop in rates of ER presentation for self-poisoning during the pandemic period, but no statistical evidence that may correlate this difference with a pre-existing psychiatric condition was found [68]. Conversely, Czeisler et al. stated that the presence of a previously diagnosed psychiatric disorder was usually associated with poorer outcomes, including suicidal ideation, during the COVID-19 pandemic [69]. Louie et al. concluded that adults with Late-Life Depression (LLD) showed a significantly higher suicidal risk during the COVID-19 pandemic [61]. Moreover, in the study by Hyland et al., several different variables associated with suicide attempts were identified, including having received treatment for a mental health disorder. Interestingly, the same study demonstrated that patients treated for mental health problems display a higher risk of engaging in NSSI when compared to people with no psychiatric history [65]. Regarding presentations of patients with a substance use disorder (SUD), there was a differential increase during the COVID-19 period that might explain the rise in suicidal ideation presentations, according to McDowell et al. Olliè et al. demonstrating how daily virtual contacts were protective factors against suicidal ideation during the first French lockdown [66], 44. For specifics about these studies, see Table 5.

## Conclusions, implications, and future directions

This study aims to provide an overview of studies investigating the relationship between suicide and COVID-19 in subjects with a pre-existing mental disorder. Results suggest that suffering from a mental disorder is a risk factor for suicidal behavior, especially during the pandemic. Some studies have also highlighted an increase in suicidal behavior that could be potentially addressable to the pandemic advent in people already affected by a psychiatric disorder. Precise diagnosis data were not clearly identifiable; however, Major Depressive Disorder outstands as a major risk factor for suicidal behavior [70], especially during the pandemic. Other psychopathological elements that stand out as risk factors for suicidality in this context are social isolation, complicated grief or loss of loved ones, loneliness, economic issues, decreased accessibility to mental health facilities, substance use disorders, alcohol abuse, autism spectrum disorder (ASD), PTSD, anxiety, fear of infection and SARS-CoV2 infection or COVID-19 disease [8, 19–21, 23–25, 28, 31–34, 37, 39, 40, 47, 49, 51, 52, 56, 63, 66]. Conversely, although most studies suggested an increase in suicidal behavior, presumably addressable to the advent of COVID-19 pandemic, disease or infection, in patients with a mental disorder, several of the works analyzed provided controversial data. Different studies did not clearly correlate mental illness and suicide risk during the pandemic but rather described the increase in suicidal behavior as a new-onset phenomenon. Although it is of utmost importance to consider that the results of the studies have several limitations; many studies included were carried out employing a cross-sectional method and could not address a direct causal relationship between suicide and COVID-19. The lack of longitudinal studies, especially on subjects with a pre-existing psychiatric condition, stands out as a limitation in obtaining specific and clarifying data. Another major inherent limitation is the reliance of most of the studies on a retrospective self-report assessment of changes in suicidal behavior. Many studies used item 9 of the Patient Health Questionnaire (PHQ) to evaluate suicide risk, which has already been shown as an insufficient assessment tool for suicide risk and ideation [71]. Moreover, a complete suicide evaluation was rarely carried out, and not all studies provided data obtained in a clinical setting. Finally, the majority of the selected studies focused on the general population, and most of the data on diagnosis was self-reported. Longitudinal studies with homogeneous samples focusing on subjects with an established diagnosis and carrying out a comprehensive physician-provided suicide assessment could yield better knowledge on this topic. Furthermore, a suicide assessment with suicide-focused scales is necessary. In conclusion, future multicenter studies with large population samples could clarify cross-country differences in suicidal behavior and risk in individuals with mental disorders.

## Data Availability

All data generated or analyzed during this study are included in this published article [and its additional information files].
